# The Effectiveness of Cognitive Behavioral Therapy on Depression and Anxiety Symptoms in Breast Cancer Patients and Survivors: A Systematic Review of Interventional Studies

**DOI:** 10.1002/brb3.70098

**Published:** 2024-10-28

**Authors:** Marzieh Azizi, Fatemeh Heshmatnia, Hamed Milani, Zohreh Shahhosseini, Leila Monjazeb Marvdashti, Zahra Behboodi Moghadam

**Affiliations:** ^1^ Sexual and Reproductive Health Research Center Mazandaran University of Medical Sciences Sari Iran; ^2^ Department of Midwifery, School of Nursing and Midwifery Shiraz University of Medical Sciences Shiraz Iran; ^3^ Faculty of Medicine Mazandaran University of Medical Sciences Sari Iran; ^4^ Food Health Research Center Hormozgan University of Medical Sciences Bandar Abbas Iran; ^5^ Department of Midwifery and Reproductive Health, School of Nursing & Midwifery Tehran University of Medical Sciences Tehran Iran

**Keywords:** anxiety, breast cancer, cognitive behavioral therapy, depression, survivors

## Abstract

**Introduction:**

Breast cancer (BC) is the most common type of cancer and the second cause of cancer‐related death among women. Psychological treatments such as cognitive behavioral therapy (CBT) have been used as an effective method in the treatment of depression and anxiety in BC patients, and their effectiveness has been approved in various studies.

**Objective:**

The present study aimed to systematically investigate the effectiveness of CBT on depression and anxiety symptoms in BC patients and survivors.

**Methods:**

Electronic databases such as PubMed, Scopus, Web of Science [WOS], ScienceDirect, Cochrane Library, and Google Scholar were systematically searched from “October 2023” to “February 2024.” The quality of the included studies was evaluated using the Critical Appraisal Skills Programme (CASP). In this study, the randomized controlled trials and quasi‐experimental studies that assessed the effectiveness of CBT, cognitive behavioral group therapy (CBGT), and the combination of CBT with other psychological studies on depression and anxiety of BC patients and survivors were reviewed.

**Results:**

Out of the 16 included studies, six used CBT, and six used CBGT. In the other four studies, a combination of CBT with other psychological interventions was implemented as an interventional program. Among 16 included studies, 14 studies showed that CBT, CBGT, and a combination of CBT with other psychological interventions significantly decreased the anxiety and depression scores among BC patients and survivors (*p* < 0.05), and only in two studies, no significant effect was observed.

**Conclusion:**

As most included studies showed the effectiveness of CBT in decreasing the depression and anxiety scores among BC patients and survivors, this study strongly suggests CBT as an effective non‐pharmacological method for the treatment of psychiatric disorders of BC patients during cancer treatments and also for BC survivors.

## Graphical abstract

This systematic review indicates that cognitive behavioral therapy is an effective method to reduce depression and anxiety among patients with breast cancer and survivors; therefore, this method can be used along with cancer treatment to improve the psychological status of these women.

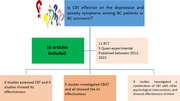

## 1 Introduction

Breast cancer (BC) is the most common type of cancer and the second cause of cancer‐related death among women (Ghaemi et al. 2019; Rodsten 2017). In Iran, BC is the most common type of cancer and the fifth cause of death due to malignancy (Jafari et al. 2018). The lifetime risk of BC is 12.5% (1 in 8 women), and the risk of death from BC is 3.6% (1 in 28 women) (Ghaemi et al. 2019). The prevalence of BC in Iranian women is reported to be 20 cases per 100,000 women (Azizi et al. 2023a; Montazeri et al. 2003). Comparing different countries, 10% of patients in developed countries and 25% of patients in developing countries are diagnosed with BC before age 40 (Azizi et al. 2023b; Zainal et al. 2013).

Although considerable advancements in BC treatment have led to better tumor response to treatments and increased life expectancy of these patients, factors such as the pain and suffering caused by the disease, worry about the family member's future, fear of death, complications caused by the disease treatment such as cancer‐related fatigue, sleep disorders, decreased function, the disturbed body image, financial and social challenges, and sexual dysfunctions lead to significant psychiatric disorders among BC patients (Bonkalo et al. 2020; Dinapoli et al. 2021; İzci et al. 2016). According to the results of two studies that examined the prevalence of psychiatric disorders in BC survivors, the prevalence of depression and anxiety was 6%–56% and 17.9%–33%, respectively (Maass et al. 2015; Zainal et al. 2013).

Although treatment with antidepressants leads to improvement of depression symptoms and quality of life (QOL) in BC patients, the evidence in this regard in BC patients has been limited, and different studies have shown different results (Andersen et al. 2013). Consumption of some selective serotonin reuptake inhibitors may reduce the effectiveness of endocrine treatments due to drug interaction with tamoxifen, which is used in patients with hormone receptor‐positive BC (Kelly et al. 2010; Ren et al. 2019); hence, attention to the effectiveness of non‐pharmacological treatments such as mindfulness‐based stress reduction (Huang et al. 2016; Zainal, Booth, and Huppert 2013), muscle relaxation intervention (Kashani et al. 2012), and cognitive‐behavioral therapy (CBT) (Getu et al. 2021; Park and Lim 2022) on psychological symptoms such as depression, anxiety, QOL, sleep problems, fear of disease recurrence, etc., in cancer patients has been increased (Lai et al. 2021; Onyedibe, Nkechi, and Ifeagwazi 2020; Sanaei et al. 2020).

Psychological treatments such as CBT have been used as an effective method in the treatment of depression and anxiety in BC patients, and their effectiveness has been approved in various studies (Karamoozian 2014; Pedram et al. 2010). A study that investigated the efficacy of CBT on the level of depression in women with BC showed that CBT, by educating adaptive coping skills to manage daily stress and encouraging the use of social resources, reduced depression levels among women with BC (Gudenkauf et al. 2015).

A literature review indicated that some systematic reviews and meta‐analyses have been conducted on the effectiveness of psychological interventions as an effective clinical method in the treatment of depression and anxiety in cancer patients (Xiao et al. 2017; Ye et al. 2018). In addition, a systematic review study has been conducted regarding the effect of CBT on anxiety and depression and the QOL of patients with early‐stage BC (Sun et al. 2019); however, there were no studies that assessed the depression and anxiety status of both BC patients and survivors; therefore, the present study aimed to systematically investigate the effectiveness of CBT on depression and anxiety symptoms in BC patients and survivors.

## 2 Materials and Methods

### 2.1 Study Design and Research Question

This systematic review was conducted according to the Preferred Reporting Items for Systematic Reviews and Meta‐Analyses (PRISMA) statements 2020 (Page et al. 2020).

Following the PICO criteria, the “participants” were women with BC or BC survivors; the type of “intervention” was CBT or cognitive behavioral group therapy (CBGT) and combined psychological interventions with CBT; the “comparison” BC patients or BC survivors received no intervention or control group, and the “outcome” was the mean score of the anxiety and depression.

According to this PICO component, the research question was formulated as follows: “Is CBT effective on the depression and anxiety symptoms among BC patients or BC survivors?” (Table 1).

**TABLE 1:** The search strategy in databases.

**Research question**	Is CBT effective on the depression and anxiety symptoms among BC patients or BC survivors?
**PICOS components**	P = Studies conducted on BC patients and survivors I = CBT, CBGT, or comparison of CBT with other psychological approaches C = Usual care or each intervention except CBT O = Depression & anxiety S = RCTs & quasi‐experimental

**Last search date**	**The latest search was performed from “October 2023” to “August 2024.”**		
**Database**	**Search strategy**	**Number of extracted studies**	**Search filters**
PubMed	(breast cancer [TIAB]) OR (breast neoplasm* [TIAB]) OR (breast tumor [TIAB]) OR (breast carcinoma [TIAB]) OR (breast cancer survivors [TIAB]) OR (cancer survivorship [TIAB]) AND (anxiety [TIAB]) OR (anxiety symptoms [TIAB]) OR (depression [TIAB]) OR (depressive symptoms [TIAB]) AND (cognitive behavioral therapy* [TIAB]) OR (cognitive behavioral group therapy* [TIAB])	1756	– Article language: English, Persian – Article type: clinical trials, randomized controlled trials, quasi‐experimental
Scopus	TITLE‐ABS‐KEY (breast cancer OR breast neoplasm OR breast tumor OR breast carcinoma* OR cancer surviv* OR cancer survivorship) AND (anxiety OR anxiety symptoms OR depression OR depressive symptoms) AND (cognitive behavioral therapy OR cognitive therapy)	253	– Subject areas: medicine – Document types: article – Source type: journal
WOS	TS = (breast cancer OR breast neoplasm* OR breast tumor OR breast cancer surviv* OR cancer survivorship) AND (anxiety OR anxiety symptoms OR depression OR depressive symptoms) AND (cognitive behavioral therapy OR cognitive behavioral group therapy)	219	– Document types: article
ScienceDirect	Breast cancer survivors OR breast cancer patients AND depression OR depressive symptoms OR anxiety OR anxiety symptoms AND cognitive behavioral therapy OR cognitive behavioral group therapy	135	– Article type: research article
Cochrane Library	Breast cancer survivors OR breast cancer patients AND depression OR depressive symptoms OR anxiety OR anxiety symptoms AND cognitive behavioral therapy OR cognitive behavioral group therapy	72	– Topic: breast cancer
Google Scholar	(“breast cancer” OR “breast neoplasm” AND “breast cancer survivors” AND “anxiety” OR “depression” OR “depressive symptoms” AND (cognitive behavioral therapy OR cognitive behavioral group therapy)	906	– Year of publication: any time – Sort by relevance – Keywords in the title of the article

### 2.2 Literature Search and Search Strategy

According to the mentioned PICO components, authors conducted comprehensive systematic searches in Google Scholar and databases including PubMed, Scopus, Web of Science, ScienceDirect, and Cochrane Library. The last search by the authors was from “October 2023” to “August 2024.” To ensure all published studies regarding this study title were retrieved, no language and publication year restrictions were considered during the search process.

To extract the keywords, the authors searched the MeSH database as follows:

“Cognitive behavioral therapy” [Mesh] OR “Cognitive behavioral therapies” [Mesh] OR “cognitive therapy” [Mesh] OR “cognition therapy” [Mesh] OR “behavioral therapy” [Mesh] OR “behavior therapy” [Mesh] AND [“depression” [Mesh] OR “depressive symptoms” [Mesh] OR “depressive disorder” [Mesh] OR “anxiety” [Mesh] OR “anxiety disorders” [Mesh] OR “anxiety symptoms” [Mesh] AND [“breast cancer” OR “breast neoplasms” [Mesh] OR “breast malignant neoplasm” [Mesh] OR “breast tumor” [Mesh] OR “breast malignant tumor” [Mesh] OR “breast carcinoma” [Mesh] OR “breast carcinomas” [Mesh] OR “survivors” [Mesh] OR “long‐term survivors” [Mesh] OR “survivorship” [Mesh] AND [(“Randomized controlled trials” [Publication Type] AND “Randomized controlled trial” [Publication Type]).

We searched each database according to the specific guidelines for advanced searches provided by each database. In the next step, the search studies reference lists were carefully checked manually to ensure that all the relevant studies were found. The search strategy for all mentioned databases is shown in Table 1. The reference manager software EndNote 21 was used to collect references and eliminate duplicate records.

### 2.3 Inclusion and Exclusion Criteria

The inclusion criteria were as follows:

All interventional studies, such as randomized controlled trials and quasi‐experimental studies, investigated the effectiveness of CBT, CBGT, or comparison of CBT with other psychological approaches on depression and anxiety in women with BC or BC survivors.

In contrast, other psychological interventions such as mindfulness, cognitive behavioral stress management, psychotherapy, psychological counseling, psychological training, psychoeducation, etc., on anxiety and depression of cancer patients, relevant articles or abstracts published in national or international conferences, study design including case reports or case series, letters to the editor and short communication, review studies such as narrative reviews, systematic reviews and scoping reviews, protocol studies, cross‐sectional and cohort studies in women BC patients and BC survivors, studies that investigated the effectiveness of CBT in patients with cancers or other cancer than breast were excluded from the study.

### 2.4 Type of Outcome Measure

The primary outcome of this study was to systematically assess the effectiveness of CBT or CBGT and the combination of CBT with other psychological interventions on depression of BC patients and BC survivors, and the secondary outcome was to evaluate the efficacy of these mentioned strategies on the anxiety of BC patients and survivors.

### 2.5 Methodological Quality Assessment

The Critical Appraisal Skills Programme (CASP) checklist assessed the quality of clinical trial studies included in this systematic review (Table 2). This tool has been widely used as a criticism tool in health‐related studies (Purssell 2020). This tool comprises nine questions, and the authors (M.A. and F.H.) independently evaluated the included studies and graded them using the CASP quality assessment criteria for RCTs. According to the strengths and weaknesses of the studies, the CASP was graded as “high,” “moderate,” and “low.” The tool generates binary scores: 1 for “satisfied” and 0 for “unsatisfied” items (Long, French, and Brooks 2020). The results of the quality assessment of the included studies showed that 15 studies had high quality, and only one had a moderate methodologic quality (Malik et al. 2022).

**TABLE 2:** Quality assessment of included studies by the Critical Appraisal Skills Program (CASP).

Reference	The study focused on the issue	Randomized assignment of patients	Did the proper selection of patients	Blinded experiment	Identified the similarity of the groups at the beginning of the trial	Treated the groups equally	Applied results in the context	Considered clinically important outcomes	Weighted the benefits over harms and cost	Quality
Getu et al. (2023)	1	1	1	0	1	1	1	1	1	High
Akkol‐solakoglu et al. (2023)	1	1	1	0	1	1	1	1	1	High
Mansouri et al. (2023)	1	1	1	0	1	1	1	1	1	High
Abbas et al. (2022)	1	1	1	0	1	1	1	1	1	High
Kousha, Shahabizadeh, and Ahi (2022)	1	1	1	0	1	1	1	1	1	High
Malik et al. (2022)	1	0	1	0	1	1	1	1	1	Moderate
Elyasi et al. (2021)	1	1	1	0	1	1	1	1	1	High
Sheikhzadeh, Zanjani, and Baari (2021)	1	1	1	0	1	1	1	1	1	High
Lai et al. (2021)	1	1	1	0	1	1	1	1	1	High
Onyedibe, Nkechi, and Ifeagwazi (2020)	1	1	1	0	1	1	1	1	1	High
Zangane Gheshlaghi et al. (2020)	1	1	1	0	1	1	1	1	1	High
Ren et al. (2019)	1	1	1	0	1	1	1	1		High
Qiu et al. (2018)	1	1	1	0	1	1	1	1	1	High
Khatibian et al. (2014)	1	1	1	0	1	1	1	1	1	High
Qiu et al. (2013)	1	1	1	0	1	1	1	1	1	High
Ghahari et al. (2012)	1	1	1	0	1	1	1	1	1	High

### 2.6 Date Extraction and Collection

The titles and abstracts of all included studies were evaluated for their relevance. At this stage, irrelevant abstracts were retained until the full text of the article was reviewed. After carefully reading the full text of the selected articles, the required information was extracted into descriptive tables and cross‐checked by M.A. Two investigators (M.A. and F.H.) independently assessed each publication for eligibility and compared the results. The final decision is based on discussions with a third reviewer (Z.B.M.) if there is a discrepancy in their assessment. The extracted data included the first author, the publication year, the country, the study design, sample size, primary outcome, intervention type, intervention duration, control group (CG) condition, outcome measurement, cancer stage, and main results of the included studies.

## 3 Results

### 3.1 Search Results

**FIGURE 1:**
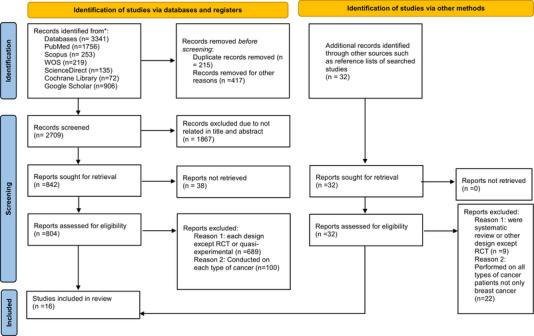
A flow chart of study selection according to PRISMA guidelines.

The systematic search resulted in 3341 articles; after removing the duplicates (*n* = 215) and records removed for other reasons (*n* = 417), 2709 studies remained. In this stage, 1867 studies were excluded based on the titles and abstracts. During the review of the full texts, a collection of articles was excluded if they were cross‐sectional, cohort, qualitative, case reports, systematic reviews, and editorial studies (*n* = 689) and conducted on each type of cancer (*n* = 100). In addition, 32 articles were identified through other sources, such as reference lists of searched studies. Among them, nine articles were systematic reviews or other designs except RCT, and 22 were performed on all cancer patients, not only BC. Finally, 16 articles were included in this systematic review (see Figure 1 for PRISMA flow diagram of the literature review).

### 3.2 Description of the Characteristics of the Included Studies

A summary of the general characteristics of the included studies is listed in Table 3. Of the 16 included studies, seven studies were conducted in Iran (Elyasi et al. 2021; Ghahari et al. 2012; Khatibian and Shakerian 2014; Kousha, Shahabizadeh, and Ahi 2022; Mansouri et al. 2023; Sheikhzadeh, Zanjani, and Baari 2021; Zangane Gheshlaghi, Shayegan Manesh, and Bankdari 2020), three in China (Qiu et al. 2018; Qiu et al. 2013; Ren et al. 2019), one in Taiwan (Lai et al. 2021), one in Nigeria (Onyedibe, Nkechi, and Ifeagwazi 2020), one in Pakistan (Abbas et al. 2022), one in Ireland (Akkol‐Solakoglu and Hevey 2023), one in Ethiopia (Getu et al. 2023), and one in Indonesia (Malik et al. 2022).

**TABLE 3:** Characteristics of included studies.

Reference	Type of trial	Sample size (IG1/CG) or (IG1/IG2/CG)	Primary outcome	Intervention type	Intervention duration	Control condition	Outcome measurement	Cancer stage	Results
Getu et al. 2023	RCT	31/31	Fatigue, depression, and QOL	CBT‐AP	Seven sessions (three 2‐h face‐to‐face and four 30‐min telephone sessions)	Usual care	BDI, CFS	I, II, III	CBT‐AP group had lower fatigue and depression scores, and higher global health status scores (*p* < 0.001).
Akkol‐Solakoglu and Hevey 2023	RCT	53/23	Anxiety and depression	Icbt	Eight weekly sessions	Usual care	EORTC‐QLQ, CWS, COPE, MOS‐SSS	—	iCBT group had lower anxiety and depression (< 0.015, *p* < 0.002), respectively.
Mansouri et al. 2023	Quasi‐experimental with a pretest/posttest	25/25	Depression, anxiety, and pain‐coping strategies	CBGT	Ten sessions of 90 min weekly	Usual care	BDI, BAI, CSQ	I, II, III	CBGT significantly decreased the mean score of depression (19.50 vs. 29.92, *p *< 0.001) and anxiety levels (19.66 vs. 28.17, *p* < 0.001) and increased the mean score of pain‐coping strategies (31.76 vs. 16.03, *p* < 0.001) in BC patients compared to CG.
Abbas et al. 2022	CT	30/30	Psychiatric comorbidity and QOL	CBT	One session weekly for 3–4 months	Waitlist	DASS, DAS, RSES	I, II	Depression, anxiety, and stress‐related symptoms significantly reduced after CBT treatment in stage I and stage II (*p < *0.001).
Kousha, Shahabizadeh, and Ahi 2022	Quasi‐experimental	15/15	Depression, cognitive flexibility, and cognitive regulation of emotion	CBGT	eight 90‐min sessions	Not reported	BDI, DVWCFQ, GSCERQ	—	CBGT increased the cognitive flexibility and cognitive regulation of emotions (*p < *0.05).
Malik et al. 2022	Quasi‐experimental	11/11	Anxiety	CBT, an educational video regarding anxiety management	Not reported	Anxiety management	STAI	—	CBT had no significant effect on reducing the level of anxiety (*p > *0.05).
Elyasi et al. 2021	CT	15/20/15	Anxiety, depression, and QOL	CBT, Hypnosis	Eight 1‐h treatment sessions	Not reported	QOL, HADS	—	In CBT and hypnosis groups, the stress differences were statistically significant (between effect, *p* = 0.001, and 0.005), depression differences in CBT and hypnosis groups were statistically significant (between effect, *p* = 0.001 and 0.002), respectively.
Sheikhzadeh, Zanjani, and Baari 2021	RCT	20/20/20	Anxiety, depression, and fatigue	CBT, MBCT	Eight 90‐min sessions	Wait list	BDI, CFS, BAI	—	CBT (*p < *0.001) and MBCT (*p < *0.001) significantly decrease the anxiety score compared to CG.
Lai et al. 2021	RCT	36/34	Depression, anxiety, and sleeping quality	CBTM	12 weekly sessions over 3 months	Usual care	PSQI, HADS,	I, II, III, IV	CBTM increases sleep quality (*p < *0.001), reduces anxiety (*p < *0.0001) and depressive symptoms (*p < *0.0001).
Onyedibe, Nkechi, and Ifeagwazi 2020	RCT	16/15	Anxiety and depression	CBGT	12 weekly 90 min over 2 months	Booklet	HADS	I, II, III	Participants in the CBGT had a significant decrease in anxiety and depression scores compared to CG (*p < *0.001).
Zangane et al. 2020	Quasi‐experimental study with pretest/posttest	20/20	Depression and anxiety	CBGT	12 weekly sessions	Not reported	BDI, STAI	—	The mean scores of anxiety and depression variables in the CBGT group in the post‐test decreased compared to the pre‐test (*p* = 0.001).
Ren et al. 2019	RCT	98/98/196	Anxiety and depression	CBT	12 weekly sessions	Usual care	HAMD, HAMA	—	Women in the CBT group showed significantly less depressive and anxiety symptoms compared to CG (*p < *0.001).
Qiu et al. 2018	RCMT	98/98/196	Depression, insomnia, and QOL	CBT	Nine sessions for 12 weeks	Usual care	AIS, FACT‐B	—	Participants in the CBT group showed fewer depression and insomnia problems and better overall QOL (*p < *0.01).
Khatibian et al. 2014	Quasi‐experimental study with pretest/posttest	12/12	Depression, anxiety, and stress	CBGT	10 weekly 90‐min sessions	Usual care	DASS‐21	I, II, III	CBGT significantly decreased the mean score of depression (27.58 vs. 29.92, *p <* 0.001), anxiety (24.08 vs 28.08, *p < *0.001), and stress (17.33 vs 27.08, *p < *0.001) in the IG compared to the CG.
Qiu et al. 2013	RCT	31/31	Anxiety and depression	CBGT	10 weeks 30‐min sessions	Waitlist	HAMD, SAS, SES	I, II, III, IV	The CBGT group had a significant reduction in depression (*p < *0.001) and no significant reduction in anxiety (*p > *0.005) scores. CBT also significantly improves the QOL (*p < *0.01) and self‐esteem (*p < *0.05) compared to CG.
Ghahari et al. 2012	RCT	15/15/15	Anxiety and depression	CBT, spiritual‐religious	Eight weekly sessions	Waitlist	BDI, BAI	—	There was no significant difference in anxiety and depression in IGs compared to CG (*p > *0.05).

Abbreviations: AIS, Athens Insomnia Scale; BAI, Beck Anxiety Inventory; BDI, Beck Depression Inventory; Brief COPE, Brief Coping Orientation to Problems Encountered; CBGT, cognitive behavioral group therapy; CBT, cognitive behavioral therapy; CBT‐AP, cognitive behavioral therapy integrated with activity pacing; CBTM, cognitive behavioral therapy plus coping management; CFS, Cancer‐Related Fatigue Scale; CG, control group; CSQ, Coping Strategy Questionnaire (Rosenstiel and Keefe); CT, clinical trial; CWS, Breast Cancer Worry Scale; DAS, Death Anxiety Scale; DASS, Depression Anxiety And Stress Scale; DVWCFQ, Dennis and Vander Wal Cognitive Flexibility Questionnaire; EORTC‐QLQ, European Organization for Research and Treatment of Cancer Quality of Life Core Questionnaire; FACT‐B, functional assessment of cancer therapy‐breast; GSCERQ, Garnefski, Kraaij and Spinhoven Cognitive Emotion Regulation Questionnaire; HAMA, Hamilton Anxiety Scale; HAMD, Hamilton Depression Rating Scale; HADS, Hospital Anxiety And Depression Scale; iCBT, internet‐delivered cognitive behavioral therapy; IGs, intervention groups; MBCT, mindfulness‐based cognitive therapy; MOS‐SSS, Medical Outcomes Study Social Support Survey; PSQI, Pittsburgh Sleep Quality Index; QOL, Quality Of Life Questionnaires; RCMT, randomized control multicenter trial; RCT, randomized control trial; RSES, Rosenberg Self‐Esteem Scale; SAS, Self‐Rating Anxiety Scale; SES, Self‐Esteem Scale; STAI, State‐Trait Anxiety Inventory.

The included studies were published from 2012 to 2023, had various sample sizes (22–392), and considered 1268 BC survivors women. Based on the type of trial, 11 of the included studies were clinical trials (Abbas et al. 2022; Akkol‐Solakoglu and Hevey 2023; Elyasi et al. 2021; Getu et al. 2023; Ghahari et al. 2012; Lai et al. 2021; Onyedibe, Nkechi, and Ifeagwazi 2020; Qiu et al. 2018; Qiu et al. 2013; Ren et al. 2019; Sheikhzadeh, Zanjani, and Baari 2021), while five studies were quasi‐experimental research (Khatibian and Shakerian 2014; Kousha, Shahabizadeh, and Ahi 2022; Malik et al. 2022; Mansouri et al. 2023; Zangane Gheshlaghi, Shayegan Manesh, and Bankdari 2020). Moreover, blinding was not mentioned in the included studies. Of the included studies, the duration of implementing the intervention was variable between eight and 12 sessions, but most of the authors had considered eight 90‐min interventional programs for their participants. Regarding the anxiety and depression measurements in the included studies, different questionnaires were used.

The included studies had applied different intervention types, so six studies used cognitive behavioral therapy (CBT) as an interventional program (Abbas et al. 2022; Akkol‐Solakoglu and Hevey 2023; Getu et al. 2023; Malik et al. 2022; Qiu et al. 2018; Ren et al. 2019). In six studies, CBGT (Khatibian and Shakerian 2014; Kousha, Shahabizadeh, and Ahi 2022; Mansouri et al. 2023; Onyedibe, Nkechi, and Ifeagwazi 2020; Qiu et al. 2013; Zangane Gheshlaghi, Shayegan Manesh, and Bankdari 2020) In other studies, a combination of CBT and hypnosis (Elyasi et al. 2021), CBT and spiritual religious (Ghahari et al. 2012), CBT plus coping management (CBTM) (Lai et al. 2021), mindfulness‐based cognitive behavioral therapy (MBCT), and CBT (Sheikhzadeh, Zanjani, and Baari 2021) had been implemented as an interventional program.

### 3.3 CBT

In six studies, the effectiveness of CBT on the depression and anxiety of BC patients and survivors was investigated. In a study by Ren et al., participants were randomly divided into CBT, self‐care management (SCM), and CG. Women in the CBT and SCM groups received nine‐session programs, whereas CG didn't receive any intervention; six CBT sessions were held, and women in the CBT group showed significantly fewer depressive and anxiety symptoms compared to SCM and CG (*p* < 0.01) (Ren et al. 2019). In another study conducted in China, participants received nine sessions of CBT and SCM for 12 weeks as an interventional program, and CG just received the usual care after the intervention. Participants in the CBT group showed fewer insomnia problems and better QOL than SCM and CG (*p* < 0.01) (Qiu et al. 2018). In a clinical trial assessing CBT on psychiatric comorbidity and QOL, the results showed that CBT had significantly reduced anxiety, depression, and stress after intervention among the intervention group compared to CG (*p* < 0.000) (Abbas et al. 2022). In a study conducted in Indonesia, the intervention group (*n* = 11) was given educational videos on anxiety management and CBT, and the CG (*n* = 11) was only given educational videos related to anxiety management. The results showed that CBT therapy did not show any significant results in reducing the patient's anxiety level compared to CG (*p* = 0.878) (Malik et al. 2022).

In this respect, in one study reflected on the effect of internet‐delivered cognitive behavioral therapy (iCBT) on depression and anxiety, 72 participants (CG = 23, iCBT = 49) were randomized to a seven‐module guided iCBT intervention weekly over 8 weeks. The results indicated that patients in the iCBT group had no significant lower depression and anxiety scores than CG (*p* = 0.075) (Akkol‐Solakoglu and Hevey 2023). Two other studies assessing CBT integrated with activity pacing (Getu et al. 2023) and CBT (Qiu et al. 2018) on depression, fatigue, insomnia, and QOL of BC patients showed effectiveness compared to the CG.

### 3.4 CBGT

In six studies, the effect of CBGT on depression and anxiety symptoms was evaluated (Khatibian and Shakerian 2014; Kousha, Shahabizadeh, and Ahi 2022; Mansouri et al. 2023; Onyedibe, Nkechi, and Ifeagwazi 2020; Qiu et al. 2013; Zangane Gheshlaghi, Shayegan Manesh, and Bankdari 2020). For example, in a quasi‐experimental study in Iran that investigated the efficacy of CBGT on depression, cognitive flexibility, and cognitive regulation of emotion, eight 90‐min sessions were held for the CBT group, and CG didn't receive any intervention. The results indicated a significant difference between the scores of the two groups in the mentioned factors (*p* < 0.05) (Kousha, Shahabizadeh, and Ahi 2022). Onyedibe et al. further considered a CBGT program for an intervention group with the aim of the effectiveness of CBT on anxiety and depression. Results indicated after two months of intervention (12 sessions), participants in the CBT group had decreased anxiety (*p* < 0.001, CI = −8.39, −2.78) and depression (*p* < 0.000, CI = −6.57) compared to CG significantly (Onyedibe, Nkechi, and Ifeagwazi 2020). Furthermore, one study compared the effect of CBGT on Chinese BC patients with major depression. The results revealed that patients in the CBGT group had a significant reduction in depression compared to CG after six months of follow‐up (*p* < 0.001). The CBGT group also yielded significantly more significant improvement than the CG concerning QOL (*p* < 0.01) and self‐esteem (*p* < 0.05). No significant differences were found between groups in improving anxiety levels (*p* > 0.05) (Qiu et al. 2013). In two quasi‐experimental studies in Iran that assessed the effectiveness of CBGT on depression, anxiety, and stress and depression, anxiety and pain coping strategies in women with BC, the results showed that this intervention significantly decreased the mean score of depression, anxiety, and stress and increased the mean scores of pain coping strategies among patients in the IG compared to the CG (*p* < 0.001) (Khatibian and Shakerian 2014; Mansouri et al. 2023). In Zangane Gheshlaghi et al., study the effectiveness of CBGT on depression and anxiety among women with BC was assessed. The results of this study showed that the mean scores of anxiety and depression in the intervention group significantly decreased compared to CG (*p* = 0.001) (Zangane Gheshlaghi, Shayegan Manesh, and Bankdari 2020).

### 3.5 Combination of CBT and Other Psychological Interventions

Four studies assessed the combination of CBT with other psychological interventions. Elyasi et al. evaluated the combination of CBT and hypnosis on anxiety, depression, and QOL of patients with BC under chemotherapy. In this clinical trial, 50 women aged 25 to 65 were assigned to three groups (hypnosis, CBT, and CG), and eight 1‐h sessions were held for each of the two intervention groups. The stress differences in the CBT and hypnosis groups were statistically significant (between effect, *p* = 0.001 and 0.005), with no significance in CG (*p* = 0.40). Moreover, depression differences in CBT and hypnosis groups were statistically significant (between effect, *p* = 0.001 and 0.002, respectively) compared to the CG (*p* = 0.40). In addition, the QOL scores improved significantly in both CBT and hypnosis groups (*p* < 0.05) (Elyasi et al. 2021).

In a clinical trial by Lai et al., the effectiveness of CBTM on depression and anxiety of BC patients was assessed. For this purpose, the CBTM intervention was implemented for the 12‐week session (*n* = 36). At the same time, CG received usual care (*n* = 34), and the follow‐up evaluation was performed after intervention (T1), 1 month (T2), and 3 months (T3). The experimental group showed significant improvement in sleep quality and anxiety at T2 (95% CI: −2.86 to −0.24, *p* = 0.02) and T3 (95% CI: −3.86 to −1.42, *p* < 0.0001) and depressive symptoms at T2 (95% CI: −2.53 to −1.08, *p* < 0.0001) and T3 (95% CI: −5.92 to −3.99, *p* < 0.0001), and significant increases in their QOL at T2 (95% CI: 2.06–9.88, *p* < 0.0001) and T3 (95% CI: 5.52–14, *p* < 0.0001) (Lai et al. 2021).

In Sheikhzadeh, Zanjani, and Baari (2021), the effectiveness of mindfulness‐based CBT (MBCT) on anxiety, depression, and fatigue was assessed. Participants were divided into three groups (MCBT, CBT, and CG), and the experimental groups received eight weekly treatment sessions. The results exhibited that anxiety decreased after intervention between two groups of MBCT and CBT (*p* < 0.001), and a significant reduction in depression was observed in both groups (*p* < 0.001); however, no significant differences in fatigue score in the intervention groups (*p* = 0.84) and CG (0.10) were seen (Sheikhzadeh, Zanjani, and Baari 2021).

A study was conducted to evaluate the effect of CBT and spiritual‐religious (SR) intervention on reducing anxiety and depression in BC patients. Accordingly, 30 women in the intervention group participated in eight CBT and SR program sessions, and the women in CG didn't receive the intervention. The results showed that although the mean scores of anxiety and depression in the intervention groups were more than CG, the difference was not statistically significant (*p* > 0.05) (Ghahari et al. 2012).

## 4 Discussion

This systematic review aimed to review the effectiveness of CBT on depression and anxiety symptoms in BC patients and survivors. The literature comprehensive search indicated that although various studies were conducted regarding the efficacy of CBT on the psychological health of patients, the assessment revealed that most of the eligible studies were conducted in East Asia. Studies conducted in European and American countries mainly assessed the patients with each cancer or assessed other outcomes except depression and anxiety, which were not included in this systematic review. The results of a study that compared the anxiety and health‐related QOL among BC patients in China and the United States showed these findings suggest that anxiety and its association with QOL among patients with BC varies depending on cultural context, which reveals the higher anxiety and poorer QOL among Chinese patients compared with US patients (You et al. 2017).

In addition, the literature review showed that various systematic reviews were conducted regarding the different psychological interventions, such as mindfulness‐based therapy or mindfulness‐based stress reduction (Castanhel and Liberali 2018; Matthews, Grunfeld, and Turner 2017; Piet, Würtzen, and Zachariae 2012; Zhang et al. 2016), psychosocial interventions (Johannsen et al. 2013), psychoeducational intervention (Al‐Alawi et al. 2022), and other psychological treatments for depression, anxiety, fatigue, sleep disorders, pain, BC recurrence, and other psychological and physical outcomes of BC patients. In addition, although various studies were performed regarding the effectiveness of CBT on the psychological status of BC patients, no systematic review was found to assess the anxiety and depression of both BC patients and survivors.

Most of the included studies’ results indicated CBT, CBGT, and a mix of CBT and other strategies had a significant effect on reducing depression and anxiety levels in patients with BC. Consistent with the results of this study, the findings of the meta‐analysis investigating the impact of CBT on QOL and the psychological health of BC patients and survivors showed that according to the results of 10 included studies, CBT had a significant effect on increasing the QOL (effect size [ES] = 0.57, 95% CI: 0.44–0.69; *p* < 0.001) and decreased the score of depression (ES = −1.11, 95% CI: −1.28 to −0.94; *p* < 0.001), anxiety (ES = −1.10, 95% CI: −1.27 to −0.93, *p* < 0.001), and stress (ES = −0.40, 95% CI: −0.53 to −0.26, *p* < 0.001) significantly. This study suggested that CBT is an effective intervention and pharmacological treatment to improve the psychological status of BC patients and survivors (Ye et al. 2018). In addition, the results of a meta‐analysis conducted to review the efficacy of psychological interventions on depression of BC patients after breast surgery showed that CBT as a common psychological intervention had a strong significant effect in reducing the depression level among patients with breast surgery; however, the need for more high‐quality studies to provide the valid results is suggested in this study (Xiao et al. 2017). The results of a systematic review that assessed the effect of CBT on the treatment of depression, anxiety, and QOL of early‐stage BC patients showed that CBT only decreased the score of anxiety among IG compared to the CG (*p* = 0.04) and no significant effect was observed on depression and QOL among patients (*p* > 0.05). Regarding depression, the results of this study were inconsistent with this current systematic review. This difference can be due to the included studies in this mentioned study being until 2017, while in this study, all of the studies published until 2024 were included and showed the effectiveness of CBT on depression and anxiety levels. In addition, all studies on BC and BC survivors were included in this systematic review, leading to different results. According to the published evidence, the CBT strategy is based on behavioral and cognitive psychology principles. It has shown positive effectiveness in the treatment of psychiatric disorders among BC patients (Xiao et al. 2017).

### 4.1 Limitations

The limitation of this study was that due to some of the studies not reporting the mean score of depression and anxiety, and also due to different measurement tools, the research team could not perform the meta‐analysis.

## 5 Conclusion

This systematic review revealed that most of the included studies showed the effectiveness of CBT or CBGT and combined psychological intervention with CBT on depression and anxiety mean scores compared to CG. The findings of this study strongly suggest the use of the CBT strategy by psychologists, psychiatrists, and other healthcare providers as an effective non‐pharmacological method for the treatment of psychiatric disorders of BC patients during cancer treatments and also for BC survivors to improve their psychological status.

## Author Contributions

**Marzieh Azizi and Fatemeh Heshmatnia**: conceptualization, methodology, writing–original draft, writing–review and editing. **Zahra Behboodi Moghadam and Zohreh Shahhosseini**: writing–review and editing, supervision and project administration. **Hamed Milani and Leila Monjazeb Marvdashti**: conceptualization, supervision, project administration, writing–review and editing, validation.

## Conflicts of Interest

The authors declare no conflicts of interest.

### Peer Review

The peer review history for this article is available at https://publons.com/publon/10.1002/brb3.70098.

## Data Availability

The data that support the findings of this study are available from the corresponding author upon reasonable request.
